# *Ac/Ds*-Induced Receptor-like Kinase Genes Deletion Provides Broad-Spectrum Resistance to Bacterial Blight in Rice

**DOI:** 10.3390/ijms23094561

**Published:** 2022-04-20

**Authors:** Qiong Mei, Yu Wen Fu, Tian Miao Li, Yuan Hu Xuan

**Affiliations:** College of Plant Protection, Shenyang Agricultural University, Shenyang 110866, China; meiqiong@syau.edu.cn (Q.M.); 2020220492@stu.syau.edu.cn (Y.W.F.); 2021220476@stu.syau.edu.cn (T.M.L.)

**Keywords:** *Ac/Ds*, chromosomal deletion, receptor-like kinase, broad-spectrum resistance, rice

## Abstract

Rice bacterial blight caused by *Xanthomonas oryzae* pv. *oryzae* (*Xoo*) seriously affects rice yield production. The discovery and application of broad-spectrum resistance genes are of great advance for disease resistance breeding. Previously, we identified that multiple *receptor-like kinase (RLK)* family gene deletions induced by the *Ac/Ds* system resulted in a lesion mimic symptom. In this study, the mutant #29 showed that this lesion mimic symptom was isolated. Further analysis identified that four *RLK* genes *(RLK19-22)* were deleted in the #29 mutant. The #29 mutant exhibited broad-spectrum resistance to *Xoo* and subsequent analyses identified that pathogenesis-related genes *PR1a, PBZ1*, and cellular H_2_O_2_ levels were significantly induced in the mutant compared to wild-type plants. A genetic analysis revealed that reconstruction of *RLK20*, *RLK21,* or *RLK22* rescued the lesion mimic symptom of the #29 mutant, indicating that these three *RLKs* are responsible for broad-spectrum resistance in rice. Further yeast two hybrid and bimolecular fluorescence complementation assays demonstrated that RLK20 interacts with RBOHB, which is a ROS producer in plants. Compared to wild-type plants, the #29 mutant was more, while #29/*RLK20* *ox* was less, susceptible to MV (methyl-viologen), an ROS inducer. Co-expression of *RLK20* and *RBOHB* reduced RBOHB-promoted H_2_O_2_ accumulation in the cells. Taken together, our research indicated that the RLKs may inhibit RBOHB activity to negatively regulate rice resistance to *Xoo*. These results provide the theoretical basis and valuable information about the target genes necessary for the successful breeding of rice cultivars resistant to bacterial blight.

## 1. Introduction

Rice is an important crop that feeds more than 50% of the world’s population. Rice bacterial blast (BB) caused by *Xanthomonas oryzae* pv. *oryzae* (*Xoo*) is a serious disease that severely threatens yield production. In the 1980s, large outbreaks of the disease were frequent [1]. The disease has been effectively controlled with the application of resistance genes such as *Xa3/Xa26* and *Xa4* during the breeding process [2,3]. More than 40 resistance genes have been identified to date and 11 of these genes have been successfully cloned [4,5]. These resistance genes encode different types of proteins. For example, *Xa3/Xa26*, *Xa4,* and *Xa21* encode receptor-like kinase; *Xa13*, *Xa25,* and *Xa41* encode sugar and are eventually exported as a transporter (SWEET); *Xa10*, *Xa23,* and *Xa27* encode executor proteins; and *Xa1* and *Xa5* encode other types of proteins [6].

*Xa3/Xa26*, *Xa4,* and *Xa21* encoding receptor-like kinase are involved in PAMP- Triggered Immunity (PTI) [3,7,8]. Both *Xa3/Xa26* and *Xa21* confer broad-spectrum resistance to various *Xoo* races [8,9]. *Xa26* was first identified in the rice *indica* variety Minghui 63 [9]. It is the same gene as *Xa3* identified in the *japonica* variety Wase Aaikolu 3 [7]. XA3/XA26 interacts with somatic embryogenesis receptor kinase 2 (OsSERK2) and triosephosphate isomerase 1.1 (OsTPI1.1) to further regulate rice resistance to *Xoo*. Suppression of *OsTPI1.1* in rice weakens its resistance to *Xoo* [10]. Similar to the role of OsTPI1.1, OsSERK2 positively regulates the rice resistance via the interaction with XA3/XA26 [11]. XA21 originated from *Oryza longistaminata*, is a transmembrane immune receptor that responds to sulfated derivatives from *Xoo,* and induces XA21-mediated immunity X (RaxX) in rice [12]. While Xa21-mediated resistance is not sustained throughout the entire growing season, the rice plants achieve full resistance only at the adult stage [13]. The XA21/RaxX interaction fits the “gene-for-gene relationship” theory, like plants without *XA21* that are susceptible to *Xoo* strains even when RaxX is produced. Furthermore, rice plants that harbor *XA21* in the genome also fail to respond to *Xoo* strains without RaxX production [14,15,16,17]. *Xa4* encodes a kinase, which belongs to the subfamily of receptor-like kinases (RLKs), which are localized on the cell wall. Unlike *Xa3/Xa26* and *Xa21*, *Xa4* is a race-specific resistance gene to *Xoo* and strengthens the cell wall during the entire growing season of rice [3,18]. *Xa4* is one of the most widely used genes in *Xoo* resistance breeding in rice, since it does not compromise yield production [3]. Incompatible interactions of rice-*Xoo* that induce *Xa4* expression further increase cellulose synthase (CesA) levels to strengthen the cell wall, leading to *Xoo* resistance. In addition, *Xa4* induces the production of phytoalexins sakuranetin and momilactone A to inhibit *Xoo* [19,20,21].

Although there have been many resistance-breeding studies over recent years, complete control of the disease remains a challenge. One important reason for the disease outbreaks may be due to the production of new toxic effectors or the loss of avirulence (Avr) function of the effector belonging to *Xoo*, which can further cause the loss of resistance of a previously resistant cultivar [22,23]. The phenomenon has been reported that, due to the large-scale cultivation of rice varieties with a single resistance background, selection pressure was increased, thus further inducing *Xoo* mutation and breakthrough variety resistance [24,25]. The polymerization of resistance genes is an effective strategy for disease resistance breeding, however, it is time-consuming. The discovery of broad-spectrum resistance genes to multiple races and elucidation of their resistance mechanisms can provide resources and a theoretical basis for disease control.

In rice, 29% of all predicted 37,544 genes are family genes clustered on the genome (International Rice Genome Sequencing Project, 2005). However, the evolutionary significance and function of these family genes remain largely unknown. Due to the redundant function of these family genes, mutation of a single gene has often failed to generate an identifiable phenotype, making gene function studies challenging. The *Ac/Ds* system [26] was put forward as an excellent tool for functional studies and germplasm innovation. The maize *Ac* (*Activator*) element encodes a transposase, which catalyzes the transposition of *Ds* (*Dissociation*) elements. In general, the transposition results in the excision of the element from a donor site and insertion into a target site. However, recognition of the 5′ and 3′ ends of different *Ac/Ds* elements by Ac transposase could induce alternative transposition events, including deletions, duplications, inversions, and other sequence rearrangements [27,28,29]. In addition, the transposition of *Ac/Ds* preferentially occurs in the genic regions, which would shuffle the coding and regulatory sequences, and thereby generate new genes [30]. The frequency of transposon-induced chromosomal rearrangements increases by at least three times than found in natural populations of maize regenerated via tissue culture [26].

In this study, *Ac/Ds*-induced chromosomal deletions at RLK locus were identified. Among the deletion mutants, *RLK* (*19–22*) mutant #29 with four *RLKs* deletions exhibited broad-spectrum resistance to *Xoo* races. PR genes and H_2_O_2_ were largely induced in the #29 mutant. Genetic analysis indicated that *RLK20*, *RLK21*, *RLK22* have redundant functions in regulating the lesion mimic phenotype of the #29 mutant. Furthermore, RLK20 was identified to interact with RBOHB. Compared to the wild-type plants, the #29 mutant plants were more, while #29/*RLK20 ox* plants were less, susceptible to MV, which is an ROS inducer. Furthermore, co-expression of *RBOHB* and *RLK20* reduced RBOHB-promoted H_2_O_2_ generation. These results indicated that the RLKs negatively regulate rice broad-spectrum resistance to multiple races of *Xoo* by controlling the H_2_O_2_ and PR gene levels. These results provide useful information for using the *Ac/Ds* system to study clustered gene families in plants through the identification of the RLK functions in rice defense.

## 2. Results

### 2.1. A Pair of Ds Elements Generate Diverse Chromosomal Rearrangements

**Figure 1:**
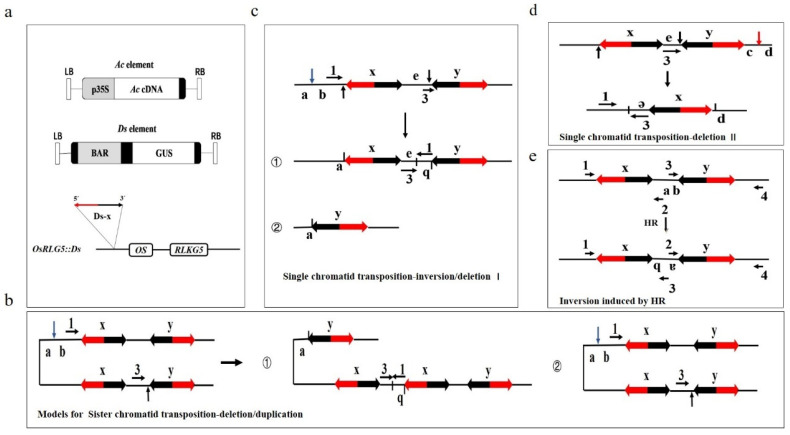
Models for transposon *Ac/Ds*-induced chromosomal rearrangements. (**a**) Models of *Ac* and *Ds* T-DNA vectors and the *OsRLG5::Ds* allele. (**b**) Models of sister chromatid transposition-deletion/duplication. (**c**) Models of single chromatid transposition-inversion/deletion I. (**d**) Models of single chromatid transposition-deletion II. (**e**) Models of the homologous recombination of the chromatid.

The amount of various family genes that display redundant function clustering at the chromosomes hampers the study of gene function in rice. *Ac/Ds* transposable elements generate chromosomal rearrangements including deletions, inversions, and duplications via the alternative transposition mechanism in rice [29]. To study the function of the redundant family genes, many chromosome fragment rearrangement/deletion mutants induced by the *Ac/Ds* system were developed. The T-DNA provided for transposase was constructed containing the *CaMV 35S* promoter to drive the *Ac* cDNA. Another T-DNA was constructed containing a modified *Ds* element (Figure 1a). A schematic diagram of the mechanism of a pair of closely located *Ds*-induced deletion/rearrangements and homologous recombination on chromosomes is presented in Figure 1 b–e. The mechanism was analyzed in detail in a previous study [28].

The 3′ and 5′ ends from two different *Ds* elements were re-inserted into the *OsRLG1-36* region after being cut by the *Ac* transposase, which is known to be the alternative transposition [29]. *OsRLG1-36* homologous genes encoding receptor-like kinases clustered on the short arm of rice chromosome 1. One line, *OsRLG5::DS*, was isolated by screening the transformed rice plants. This line possessed a single copy *Ds* insertion in the promoter region of OsRLG5 (Receptor Like Kinase Gene 5) (Figure 1a), which is related to *Leaf rust resistance 10* (*Lr10*).

### 2.2. Ac/Ds-Induced RLK Deletion Mutants Exhibited Broad-Spectrum Resistance to Xoo

**Figure 2:**
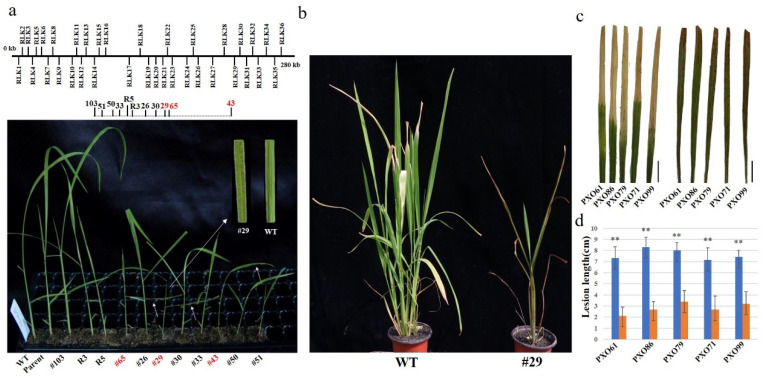
The #29 mutant plants have broad resistance to *Xoo*. (**a**) The #29 mutant was generated by the *Ac/Ds* system. WT, wild-type plants (Dongjin). (**b**) The #29 mutant and wild-type plant (Dongjin). (**c**) The leaves of #29 and wild-type plant after inoculation with *Xoo* strains (PXO61, PXO86, PXO79, PXO71 and PXO99). (**d**) Lesion length of wild-type plant and the #29 mutant after inoculation of *Xoo* strains (PXO61, PXO86, PXO79, PXO71 and PXO99). The lesion length in wild-type and the #29 plants was calculated. Data indicates average ± standard error (SE) (*n* > 6). **, *p* < 0.01.

In a previous study, we identified a *Ds* element at the *RLK19/RLG5* locus. We found that this *RLK* family consists of 36 members and contains a cluster located on chromosome 1 [29]. To test the *RLK* family function, two *Ds* elements closely located at the *RLK19* locus were first generated and the large fragment deletions with one to 11 *RLKs* deletions were further identified. Among them, the deletion mutants with a loss of eight and 11 *RLKs* exhibited lesion mimic symptoms. In this study, the deletion lines were further analyzed and the #29 mutant with the *RLK19-22* deletion was identified (Figure 2a). The #29 mutant displayed lesion mimic symptoms (Figure 2b). To analyze whether the #29 mutant had the characteristics of autoimmunity, mutant plants were first inoculated with five different *Xoo* races (PXO61, PXO71, PXO79, PXO86, PXO99). The lesion lengths were measured two weeks after the inoculation and the results demonstrated that the #29 mutant plants exhibited high and broad-spectrum resistance to *Xoo* (Figure 2c,d).

### 2.3. RLK20, RLK21, and RLK22 Regulate the Broad-Spectrum Resistance to Xoo in Rice

**Figure 3:**
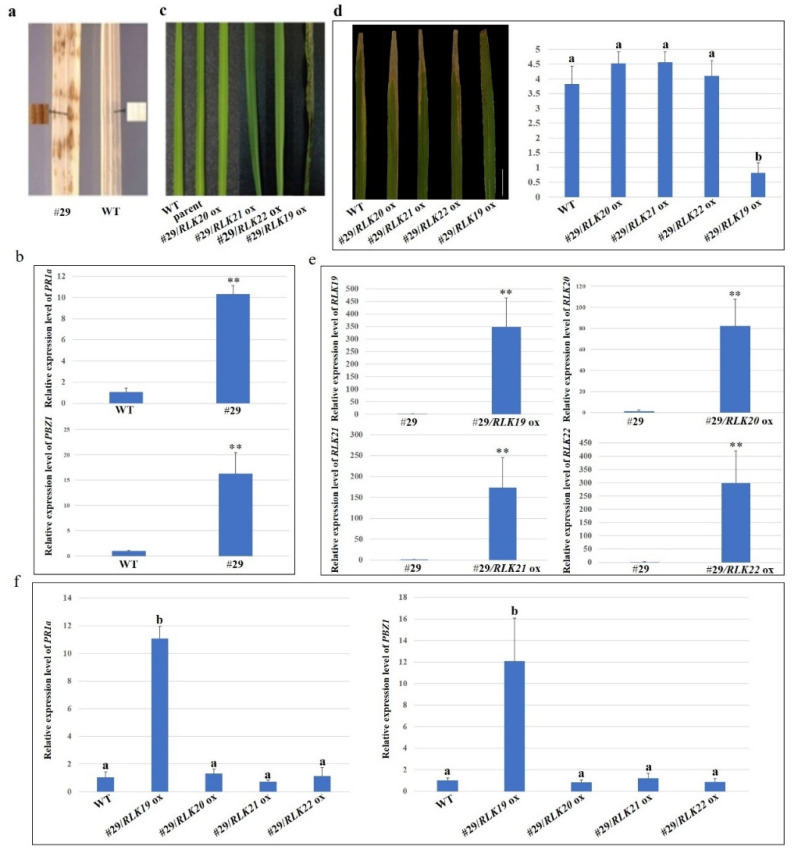
*RLK20*, *21,* and *22* can rescue the lesion mimic symptoms of the #29 mutant plant. (**a**) DAB staining of the #29 mutant and wild-type leaves. (**b**) The relative expression levels of *PR1a* and *PBZ1* in WT (wild-type plants) and the #29 mutant plants. Data indicates the average ± standard error (SE) (*n* > 6). **, *p* < 0.01 (**c**) The leaves of the WT (wild-type) plant, parent (plants with two *Ds* do not have alternative transposition on chromosome), #29/*RLK19* ox, #29/*RLK20* ox, #29/*RLK21* ox and #29/*RLK22* ox plants. #29/*RLK19* ox, #29/*RLK20* ox, #29/*RLK21* ox and #29/*RLK22* ox indicate over-expression of *RLK19*, *20*, *21,* and *22* in the #29 mutant. (**d**) Lesion length on leaves after inoculation with PXO86. Data indicates average ± standard error (SE) (*n* =10). The letters a and b denote significant differences. *p* < 0.01. (**e**) The relative expression levels of *RLK19*, *20*, *21,* and *22* in *#29/RLK19* ox, *#29/RLK20* ox, *#29/RLK21* ox, and *#29/RLK22* ox plants, respectively. Data indicates average ± standard error (SE) (*n* > 6). **, *p* < 0.01 (**f**) Relative expression levels of *PR1a* and *PBZ1* in WT, *#29/RLK19*, *#29/RLK20*, *#29/RLK21,* and *#29/RLK22 OX* plants. Data indicates the average ± standard error (SE) (*n* > 6). The letters a and b denote significant differences. *p* < 0.01.

DAB staining showed that the level of H_2_O_2_ was significantly higher in the #29 mutants compared to the wild-type plants (Figure 3a). In addition, the qRT-PCR results showed that relative expression levels of PR genes (*PR1a* and *PBZ1*) were significantly induced in #29 compared to those of the wild-type (Figure 3b). To investigate which *RLK* regulates lesion mimic and defense against *Xoo* in the #29 mutants, *RLK19*, *RLK20*, *RLK21,* or *RLK22* genes were individually expressed using the non-specific promoter 35S in the #29 mutant plants. The reconstruction of *RLK20*, *RLK21,* or *RLK22* all complemented the mutant phenotype, with *RLK19* proving to be the exception (Figure 3c). Leaves of the four kinds of transgenic complementary plants were inoculated with PXO86. The results showed that #29/*RLK20* ox, #29/*RLK21* ox, and #29/*RLK22* ox plants were susceptible to PXO86, similar to the wild-type plants (Figure 3d). The #29/*RLK19* ox plants, however, were resistant to PXO86. qRT-PCR was performed to detect the gene levels in the complementation plants. The qRT-PCR results demonstrated that *RLK19*, *RLK20*, *RLK21,* and *RLK22* were highly expressed in leaves, while no transcripts were detected in the #29 mutant plants (Figure 3e). The expression levels of *PR1a* and *PBZ1* in #29/*RLK20* ox, #29/*RLK21* ox, and #29/*RLK22* ox plants were similar to the wild-type plants (Figure 3f). The #29/*RLK19* ox plants, however, demonstrated significantly higher expression compared to the wild-type. These results suggest that *RLK19*, *20*, and *21* are required for the lesion mimic and bacterial resistance in the #29 mutant plants.

### 2.4. RLK20 Interacts with RBOHB to Modulate ROS Generation

**Figure 4:**
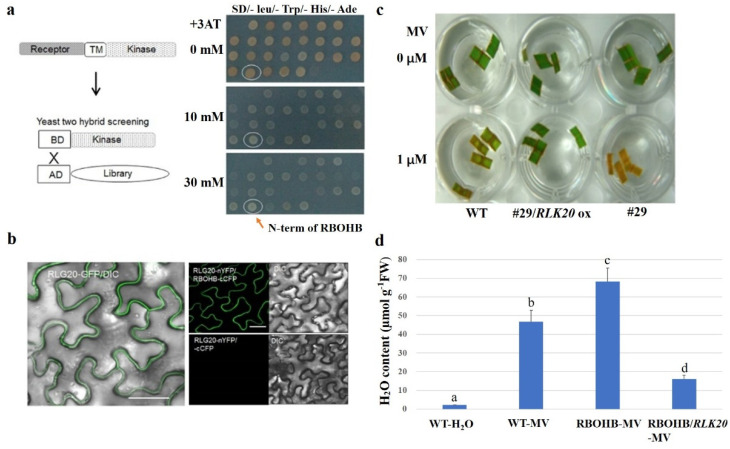
RLK20 interact with RBOHB regulating ROS production. (**a**) Screening of RLK20 interacting protein by the yeast two-hybrid system. (**b**) Colocalization of RLK20 and RBOBH in tobacco leaves by BiFC. (**c**) The leaves of the #29 mutant, #29/*RLK20 ox,* and wild-type plants after MV treatment. (**d**) H_2_O_2_ content in *RBOHB*-transformed or *RBOHB/RLK20*-transformed tobacco leaves after being treated with MV. Data indicates the average ± standard error (SE) (*n* > 6). The letters a, b, c, and d denote significant differences. *p* < 0.05.

To analyze the function of RLKs, RLK20 was selected as bait for the isolation of the interacting proteins via a yeast-two hybrid (Y2H) screening. Y2H was performed to isolate the interacting protein using the kinase domain of RLK20. Among more than 20 interactors screened, one interactor was RBOHB (Figure 4a). Sequencing of the AD-RBOHB clone identified an RBOHB fragment that contained only the N-terminal cytosolic part of the protein. To further examine the RLK20 and RBOHB interaction, the full-length RBOHB and RLK20 were analyzed in the bimolecular fluorescence complementation (BiFC) system. The results showed that RLK20 and RBOHB interacted at the plasma membrane (Figure 4b). Since *RBOHB* encodes NADPH oxidase, which catalyzes ROS production, the ROS levels in #29/*RLK20 ox*, #29 mutant, and wild-type leaves were tested using a 1µM MV treatment. The results indicated that the #29 mutant was more, while #29/*RLK20 OX* was less, sensitive to MV compared to wild-type plants (Figure 4c). Expression of *RBOHB* induced H_2_O_2_ accumulation, while co-expression of *RBOHB* and *RLK20* reduced *RBOHB*-promoted H_2_O_2_ accumulation (Figure 4d). These results indicated that RLK20 interacts with and inhibits RBOHB to reduce ROS generation.

## 3. Discussion

Rice bacterial blight seriously threatens yield production [1]. Resistance breeding is an economically and eco-friendly way to protect crops from disease. A single resistance gene can easily induce genetic mutations in the pathogen due to the widespread growing regions of rice varieties, leading to the loss of resistance in rice varieties. The discovery of resistance genes and functional studies are the basis of durable disease control. The rice genome sequencing data demonstrate that 29% of the genes are predicted to be organized in clustered gene families (International Rice Genome Sequencing Project 2005), posing a challenge in the examination of the functions of the gene families. Furthermore, functional dissection or annotation of these clustered gene families may be of significance for use in future breeding.

Previously, we identified rice plants with eight receptor-like gene (*RLK19-26*) deletions (from a clustered gene family consisting of 36 *RLKs*), by a pair of closely located *Ds* transposable elements, exhibiting lesion mimic symptoms [26]. To further investigate which *RLK* was responsible for the lesion mimic symptoms, more deletion lines were isolated to identify the chromosomal regions. Eventually, the #29 deletion mutant showed the lesion mimic symptom was isolated. Similar to other lesion mimic mutants reported [31,32,33,34,35], the growth of #29 was seriously inhibited. Further analysis identified that four *RLKs* (*RLK19-22*) were deleted in the #29 mutant. Furthermore, RLK19 shared a 39% sequence similarity with barley Lr10 [29], a leaf rust-resistant gene, implying its potential function in plant defense. The inoculation of five different races of *Xoo* strains demonstrated that the #29 plants were broad-spectrum resistant mutants. These results are similar to a previous report that identifies the broad-spectrum resistance symptoms of lesion mimic mutants [34,36]. These mutants have characteristics of chlorophyll degradation, H_2_O_2_ accumulation and apoptosis, which affect growth and development [33,34,36,37]. Furthermore, the individual *RLK* functions in the #29 mutants were analyzed by the overexpression of each *RLK* in the #29 background to ensure that the lesion mimic phenotype was caused by the loss of *RLKs*. Reconstruction of *RLKs* rescued the lesion mimic phenotype of the #29 mutant, while the *RLK19* did not rescue the mutant phenotype, indicating that three *RLKs* (*RLK20-22*) negatively regulate rice broad-spectrum resistance.

A previous study reported that *PR* gene expression levels and H_2_O_2_ content are significantly higher in the lesion mimic mutant compared to wild-type plants [34,38,39]. Similar to other lesion mimic mutants, *PR* genes (*PBZ1* and *PR1b*) expression levels and H_2_O_2_ content was significantly higher in the #29 mutants compared to wild-type plants. Interestingly, further yeast-two hybrid screening using RLK20 kinase domain as bait, identified that RBOHB (a ROS biogenesis enzyme) interacts with RLK20. MV (ROS inducer) treatment and subsequent DAB staining results showed that the #29 mutant contained more, while *#29/RLK20 ox* contained less H_2_O_2_ compared to wild-type plants. These results suggest that RLK20 may interact with RBOHB to inhibit its function. To further confirm this hypothesis, RLK20 and RBOHB were expressed in tobacco leaves, and the H_2_O_2_ level was monitored. The results showed that RLK20 expression inhibited RBOHB-mediated ROS production, implying that RLK20 may phosphorylate RBOHB to inhibit ROS generation. Previous studies demonstrated that the calcium-dependent protein kinase (CDPK) and Rac/ROP small GTPase Rac1 interact with RBOHB to activate ROS production [40,41]. RLKs may inhibit CDPK or Rac1 binding to RBOHB to reduce ROS production. However, further studies are required to explore the function of RLKs in ROS production. Alternatively, the loss of *RLK20-22* may activate RBOHB-mediated ROS production to highly accumulate the ROS, by which cell death and subsequent lesion mimic symptom were produced. Further studies are required to elucidate this issue.

Isolation and utilization of resistance-related genes is an efficient way to control the disease. However, technical limitations and the complexity of the plant genome made the resistant gene isolation task even more difficult. Diverse genetic approaches have been developed, which significantly accelerate the speed of functional genomic analysis. Due to the functional redundancy, elucidation of the clustered family gene functions is still challenging. The current study analyzed the clustered RLK family functions by *Ds*-induced chromosomal deletions and proposed the potential for using *Ac/Ds*-induced deletions as a tool for future investigations of clustered gene family functions. Taken together, we identified the RLK functions in rice broad-spectrum resistance providing target genes for future resistant cultivar breeding.

## 4. Materials and Methods

### 4.1. Tissue Culture Regeneration and Transgenic Plant Generation

The *Ac* and *Ds* gene trap cassettes were developed according to previously published methodology [42]. *Ac* and *Ds* elements were cloned into a T-DNA vector pSB11 (Figure 1a) and transformed into LBA4404 cells [29]. The tissue culture regeneration was conducted according to previously described methods [31,43]. Briefly, seeds were hulled and sterilized with 0.6% H_2_O_2_. Tissue culture media were used to produce plantlets. The regenerated plants were transplanted into bottles with solid 0.5× MS medium. The plants were then transferred to the greenhouse at 28 °C.

For the functional validation of each of the *RLK19*, *20*, *21,* and *22* genes, the entire ORFs were cloned and connected to the *pCAMBIA1381-Ubi* vector. The recombination vectors were then transformed into the rice cultivar Dongjin via *Agrobacterium tumefaciens*-mediated methods [44]. Primers designed by Primer Premier 5 for genes cloning were as follows in Table 1:

### 4.2. Rice Cultivation and Xoo Inoculation

Top second leaves of two-month-old rice plant were inoculated with five *Xoo* strains (PXO61, PXO71, PXO79, PXO86, PXO99, from Zhejiang Academy of Agricultural Sciences) using the leaf-cutting method according to a previous report [45]. The inoculated plants were stored on a plastic-covered shelf to keep moist for three days. To activate the *Xoo* strains before inoculation, the strains were inoculated on a potato semisynthetic agar (PSA) plate at 28 °C. The mature colony was further inoculated in 20 mL fluid PSA medium in a 50 mL centrifuge tube, shaken at 220 rpm until the bacterial suspension was ready for use when the OD_600_ = 0.5–1.0. The length of the disease spots from the top second leaves from six different plants was measured two weeks after the inoculation.

### 4.3. Determination of H_2_O_2_ Content

The entire ORF of *RLK20* and *RBOHB* were separately cloned into the pCAMBIA1302. *RBOHB* and *RLK20* + *RBOHB* were next injected into tobacco leaf via *A. tumefaciens*-mediated transformation. The tobacco leaves from different treatment groups were treated with 1 µM MV (Sigma, St Louis, USA) or sterile water (negative control) for 4 h. The leaves treated with MV were rinsed with sterile water, then the excess moisture on the leaves was absorbed by filter paper. The hydrogen peroxide levels of the leaves were determined according to the previously described methods [46]. Leaf tissues (1 g) were homogenized in an ice bath with 10 mL 0.1% (*w*/*v*) trichloroacetic acid (TCA). The homogenate was first centrifuged at 12,000× *g* for 15 min and then 1 mL of the supernatant was removed to 1 mL of 10 mM potassium phosphate buffer (pH 7.0) and 2 mL of 1 M (*w*/*v*) KI. The blank control consisted of 0.1% TCA without leaf extract. After the reaction was developed for one hour in darkness, the absorbance of the supernatant was measured at 390 nm by spectrophotometer. The content of H_2_O_2_ was calculated using a standard curve prepared with known concentrations of H_2_O_2_.

### 4.4. qRT-PCR Analysis

Rice leaves were collected for total RNA extraction using TRIZol reagent (Takara, Dalian, China). For qRT-PCR, RNA was reverse-transcribed to cDNA using the PrimeScript RT reagent Kit (Takara, Dalian, China) and the relative expression levels of different genes were detected using Ssofast EvaGreen Supermix (BIO-RAD, Hercules, CA, USA) with Mx3005P (Agilent, Palo Alto, CA, USA) [47]. Three technical replicates for each sample in the experiment were performed. Ubiquitin was used as an internal reference gene. All qRT-PCR primers in Table 2.

### 4.5. DAB Staining Assay

To detect H_2_O_2_ levels, rice leaves were treated with 1 µM MV or sterile water (negative control) for 24 h and stained with diaminobenzene (DAB) according to a previously published method [48]. The leaves were cut into 2 cm pieces and placed in DAB solution and incubated in a growth chamber with 25 °C for 8 h. The leaf sections were examined by light microscopy. The areas where H_2_O_2_ production occurred were reddish-brown.

### 4.6. Yeast Two-Hybrid Screening

The yeast two-hybrid screening was conducted according to the Library Construction and Screening Kits (Clontech, Dalian, China) instructions. The RLK20 kinase domain sequences were cloned into pGBKT7 as a bait vector. *RLK20*-pGBKT7 and cDNA-pGADT7-Rec or lam-pGADT7 (negative control) were co-transformed into Y2H gold yeast strain for library screening. Yeast transformants were grown on synthetic dropout-Leu-Trp-His-Ade plates.

### 4.7. BiFC Assay

*RLK20* and RBOHB were cloned into PXNGW and PXCGW, respectively [49]. For the Bi-FC assay, *RLK20*-nYFP and *RBOHB*-cCFP or cCFP (negative control) were transformed into tobacco leaves via *Agrobacterium*-mediated transformation [44]. Fluorescence of tobacco leaf was observed using a fluorescence microscope Olympus X1000. The entire *RBOHB* ORF was synthesized by Sangon Biotech company (China). The primers used for *RLK20*-nYFP were as follows: Forward, 5′-GGGGACAAGTTTGTACAAAAAAGCAGGCTTC ATGGCGATCCCTGGTTCG-3′; Reverse, 5′-GGGGACCACTTTGTACAAGAAAGCTGGGTGCTCATCCTCCTCTAAGATTTCA.

## Figures and Tables

**Table 1:** Primers used for genes clone.

Gene	LOC Number	Direction	Sequence
*RLK19*	LOC_Os01g02570	Forward	AAGCTTATGGCGATTCCTGGAGC
		Reverse	GGTACCCTAGTTACTAGCGAATTCAATTG
*RLK20*	LOC_Os01g02580	Forward	GAGCTCATGGCGATCCCTGGTTCG
		Reverse	GTTAACTCACTCATCCTCCTCTAAGATTTCA
*RLK21*	LOC_Os01g02590	Forward	AAGCTTATGGCGATTCATGGTGTGTTTC
		Reverse	GGTACCTCAACAGAAACCTGCAATCATCTTC
*RLK22*	LOC_Os01g02600	Forward	AAGCTTATGGACTTCACCAACCTTCTTATCA
		Reverse	GTTAACCTAAATCACAAGTTGATTTTGAGACG

**Table 2:** Primers used for qRT-PCR.

Gene	LOC Number	Direction	Sequences
*PR1a*	LOC_Os07g03710	Forward Reverse	GTGGGTGTCGGAGAAGCAGTG CGGCGAGTAGTTGCAGGTGAT
*PBZ1*	LOC_Os12g36880	Forward Reverse	TGGTCCGGGCACCATCTA CGAGCACATCCGACTTTAGG
*RLK19*	LOC_Os01g02570	Forward Reverse	TTGTATCAGACAGGGCATTA CCAGCCATCTCAAGTAGC
*RLK20*	LOC_Os01g02580	Forward Reverse	ACGCAATTACTGGAAGATAA TGCCTGGAAGGAGAACAC
*RLK21*	LOC_Os01g02590	Forward Reverse	CCGATGACAAGGCTACAA GAAGAGGGCAACTGCTAG
*RLK22*	LOC_Os01g02600	Forward Reverse	GTGAGTGGGAGGAGGAAC GCACCATAACGCTACAATA

## Data Availability

The data presented in this study are available on request from the corresponding author.
